# Effect of Bilateral Subthalamic Nucleus Deep Brain Stimulation on Pisa Syndrome in Parkinson's Disease

**DOI:** 10.3389/fneur.2021.739298

**Published:** 2021-10-21

**Authors:** Jing He, Zhuang Cui, Shuhua Li, Haibo Chen, Wen Su

**Affiliations:** ^1^Neurology Department, National Center of Gerontology, Beijing Hospital, Beijing, China; ^2^Neurosurgery Department, National Center of Gerontology, Beijing Hospital, Beijing, China

**Keywords:** Parkinson's disease, postural abnormality, Pisa syndrome, deep brain stimulation, subthalamic nucleus

## Abstract

**Objective:** To observe the efficacy of bilateral subthalamic nucleus deep brain stimulation on Pisa syndrome in patients with Parkinson's disease.

**Methods:** A total of 52 patients with Parkinson's disease who underwent deep brain stimulation in Beijing Hospital from July 1, 2016 to July 1, 2020 were reviewed. The clinical data were collected for the patients who met the diagnostic criteria of Pisa syndrome on “Medication-Off” state pre-operatively.

**Results:** Two patients met the diagnostic criteria of Pisa syndrome before operation, with a Pisa angle of 10 and 14°, respectively. The lateral trunk flexion of the two patients improved after operation. In stimulation-on/medication-off state, the Pisa angle decreased from 10 to 2° and from 14 to 6°, respectively.

**Conclusion:** Bilateral subthalamic nucleus deep brain stimulation might have beneficial effects on lateral trunk flexion in PD patients, but the predictors of curative effect are not clear.

## Introduction

Pisa syndrome (PS), scoliosis, camptocormia, and anterocollis are considered to be major postural abnormalities of Parkinson's disease (PD). PS is defined as trunk lateral flexion more than 10 or 15° and can be improved by passive movement or supine (1). It is reported that the incidence of PS in PD patients is 1.9–8.8% in Europe (2). A study on the prevalence and clinical characteristics of PS in 2167 Chinese patients with Parkinson's disease showed that the incidence of PS was 3.6% if Pisa angle was defined as more than 10°. The occurrence of PS was associated with older age, higher levodopa equivalents, and more severe dyskinesia (3). The majority of PD patients with Pisa syndrome are chronic onset and no obvious causes. Only a small number of PD patients develop Pisa syndrome in the form of subacute onset, which are generally related to the adjustment of medication.

PS represents a major source of functional disability for PD patients. The therapies of PS mainly focused on drug adjustment, botulinum toxin injection, and rehabilitation exercise (2). The treatment methods are relatively few and the therapeutic effect is uncertain. Deep brain stimulation (DBS) can improve motor symptoms such as tremor, rigidity, and bradykinesia in patients with PD. Current researches about DBS effect on postural abnormalities in PD are limited and mainly focused on camptocormia rather than Pisa syndrome (4, 5). We review the pre-operative information and post-operative follow-up data of all PD patients who underwent DBS in our center over a 4-year period to observe DBS effect on Pisa syndrome in Parkinson's disease.

## Methods

### Subjects

A total of 52 consecutive patients with PD who underwent DBS in Beijing Hospital from July 1, 2016 to July 1, 2020 were enrolled. All patients were diagnosed as “clinically diagnosed Parkinson's disease” according to MDS Clinical Diagnostic Criteria for Parkinson's Disease. Most patients had suffered from significant motor complications from levodopa such as motor fluctuation and dyskinesia. Pre-operative evaluation, post-operative follow-up, and programming were performed by neurologists. Also, surgery was performed by the same neurosurgeon team. All patients signed an informed consent form.

### Evaluation of Motor Symptoms and Pisa Angle

All patients were evaluated of motor symptoms and Pisa angle at two time points: baseline (1 week before the implantation of the DBS electrodes) and follow-up (9–24 months after surgery). At each time point, two different medical states were assessed as follows: At baseline, the patients were evaluated in Med-Off state (after 12-h withdrawal of levodopa and up to 72 h of other anti-Parkinson medication) and Med-On state (60 min after intake of 1.5 times the regular levodopa dose). At follow-up, assessments were performed under Med-Off/Stim-On and Med-On/Stim-On conditions.

Motor symptoms were evaluated by the third part of unified Parkinson's disease rating scale revised by movement disorder society (UPDRS-III). The Pisa angle was assessed using the following method.

All patients were photographed in the frontal upright position in each medical treatment condition at baseline and follow-up. Thus a total of 208 photos were introduced into the program NeuroPostureApp (http://www.neuroimaging.uni-kiel.de/NeuroPostureApp/) to analyze the angle of lateral flexion of the trunk. The lateral deviation (Pisa angle) was defined as the angle between a line between the midpoint of the feet and the pubic symphysis and a line between the pubic symphysis and the jugulum (4). The diagnostic standard of Pisa syndrome in this study is defined as a Pisa angle more than 10° at baseline in Med-Off state and the lateral deviation could be alleviate by supine.

### Clinical Data Collection

For patients who were consistent with the aforementioned diagnostic criteria of Pisa syndrome, we collected information on the following four aspects: (1) clinical/demographic data—gender, age of onset, PD and PS duration at DBS-implantation, the laterality of PD symptom onset, and the direction of the deviation of PS; (2) pre-surgical clinical data—UPDRS-III score of Med-Off and Med-On state, Pisa angle of Med-Off and Med-On state, and levodopa equivalent dosage; (3) operation data—DBS implantation time and implantation target; (4) post-surgical clinical data: follow-up time, DBS parameters, UPDRS-III score of Med-Off/Stim-On and Med-On/Stim-On state, Pisa angle of Med-Off/Stim-On and Med-On/Stim-On state, and levodopa equivalent dosage.

All subjects signed informed consent forms. This study was approved by the Ethics Committee of Beijing Hospital.

## Results

Of the 52 PD patients who underwent DBS, 2 met the diagnostic criteria of Pisa syndrome mentioned previously before operation.

Case 1 was a 45-year-old man with a 6-year history of PD. The first symptom was stiffness and lack of movement of the left lower limb, then the right limb was involved, and rest tremor occurred in the extremities. Madopar was effective, but the wearing-off appeared 5 years after onset. Freezing and trunk tilting to the left with low back pain appeared shortly after. The aforementioned axial symptoms were levodopa responsive. The freezing phenomenon and left tilt of the trunk may improve with Madopar, but worsen at the end of the dose. We used a levodopa challenge test to evaluate the levodopa responsiveness. At baseline Med-Off state, the score of UPDRS-III was 59, the item 28 of the UPDRS scored 3, and the Pisa angle was 10°. After administration of a levodopa challenge dose (1.5 times the usual morning dose), the score of UPDRS-III remarkably decreased to 26, item 28 of the UPDRS scored 0, and Pisa angle ameliorated to 2°.

Bilateral subthalamic nucleus (STN) DBS was performed and initial programming started 1 month after operation. The initial parameters were as follows: R-STN (monopolar configuration, cathode on contact 4) amplitude 2.2 V, pulse width 60 μs, frequency 130 Hz, L-STN (monopolar configuration, cathode on contact 7) amplitude 1.5 V, pulse width 60 μs, and frequency 130 Hz. The UPDRS-III score decreased to 20 points and the Pisa angle ameliorated to 2° in the Med-Off/Stim-On state. Six months after surgery, the stimulation of R-STN was changed to bipolar mode because of the tightness and numbness of the left limb. During the 10 months of follow-up after operation, the parameters were as follows: R-STN (bipolar configuration, cathode on contact 4, anode on contact 3) amplitude 2.5 V, frequency 130 Hz, pulse width 60 μs, L-STN (monopolar configuration, cathode on contact 7) amplitude 2.1 V, pulse width 60 μs, and frequency 130 Hz. The UPDRS-III scores were retained 20 points and the Pisa angle was 2° in the Med-Off/Stim-On state.

Case 2 was a 55-year-old man with a PD duration of 9 years. The first symptom was inflexibility and stiffness of the right limb; the left limb was involved 3 years later. His trunk tilted to the left and the wearing-off appeared in the seventh year. In pre-operative levodopa challenge test, the total UPDRS-III score reduced by up to 35.2% (from 71 to 46) after administration of 375 mg Madopar. However, item 28 of the UPDRS and Pisa angle remained unchanged regardless of being in Med-On or Med-Off state. The patient consistently maintained a 14° left lean with or without medication.

Deep brain stimulation was performed with bilateral STN. In initial programming and 6-month follow-up, UPDRS-III score reduced to 55 points in the Med-Off/Stim-On state, but the Pisa angle was still 14° with no improvement. We did further programming and increased the amplitude. The parameters were as follows: R-STN (monopolar configuration, cathode on contact 3) amplitude 2.5 V, pulse width 60 μs, frequency 130 Hz, L-STN (monopolar configuration, cathode on contact 7) amplitude 2.8 V, pulse width 60 μs, frequency 130 Hz. Fifteen months after operation, a further decrease of UPDRS-III score was observed with 43 points and the Pisa angle reduced to 6° in Med-Off/Stim-On state (see Table 1).

**Table 1:** Clinical features and outcomes in 2 patients with Pisa syndrome.

**No**.	**Disease duration at DBS (years)**	**Disease duration at PS (years)**	**Side of PD symptoms at onset**	**PS inclination**	**UPDRS-III score/Pisa angle**		**LED (mg)**	
					**Pre-surgical clinical data**	**Post-surgical clinical data**	**Pre-surgical**	**Post-surgical**
1	6	5.5	Left	Left	Med-Off 59/10° Med-On 26/2°	Med-Off/Stim-ON 20/2° Med-On/Stim-ON 18/2°	525	350
2	9	7	Right	Left	Med-Off 71/14° Med-On 46/14°	Med-Off/Stim-ON 43/6° Med-On/Stim-ON 39/6°	1,250	750

*LED, levodopa equivalent dosage.

## Discussion

Pisa syndrome (PS) is a postural deformity characterized by marked and reversible lateral trunk flexion. In this paper, we reviewed PD patients admitted to our center for DBS surgery in the past 4 years, and found that two of them had pre-operative trunk lateral flexion and met the diagnostic criteria for Pisa syndrome. These two patients had no drug adjustment before the appearance of PS. They developed Pisa syndrome 5.5 and 7 years after the onset of PD, respectively, which was consistent with a previously reported study (2).

Both patients underwent DBS surgery because of motor fluctuation. After surgery, we found their Pisa syndrome improved as effectively as their tremor and rigidity in both Med-On and Med-Off states. We therefore briefly summarize the current status regarding the treatment of Pisa syndrome with DBS.

At present, there are only 20 cases of Pisa syndrome treated by DBS in patients with Parkinson's disease (see Table 2). Among them, only one case reported by Ricciardi (8) performed DBS merely for the purpose of improving PS, and the other 19 cases underwent the surgery for motor fluctuations. Seventeen of the 20 patients were from three retrospective studies (9–11). The therapeutic targets of these three studies were bilateral STN. Fourteen patients achieved varying degrees of efficacy, while three patients with a pre-operative postural score of 3–4 on UPDRS-III item 28 in the Umemura (9) study had no improvement in both short term and long term after DBS. The other 3 of the 20 patients were case reports. Anderson (6) reported a patient's Pisa angle that decreased from ~45 to 25° with bilateral globus pallidus internus stimulation and maintained the effect for at least 4 years. Shih (7) and Ricciardi reported unilateral pedunculopontine nucleus (PPN) DBS for management of PS in patients with PD. The patients reported by Ricciardi only achieved the effect within half a year after operation, suggesting that attention should be paid to the long-term effect of this target. Interestingly, the electrodes respectively implanted in ipsilateral or contralateral PPN to the tilting side. The author explained that either ipsilateral or contralateral PPN DBS could improve PS because of the diffuse bilateral projections of PPN and its connection with the suprasegmental structures.

**Table 2:** Summary of studies for patients with parkinsonian Pisa syndrome treated with DBS.

**References**	**Study design**	**Sample size**	**Gender**	**Age (years)**	**PD duration (years)**	**PS duration (years)**	**Side of PD symptoms at onset**	**PS inclination**	**DBS target**	**Outcomes**
Anderson et al. (6)	Case report	1	Male	73	11	6	Left	Right	BL Gpi	Pisa angle decreased from 45 to 25°
Shih et al. (7)	Case report	1	Female	62	6	1	Right	Right	Left PPN	Better
Ricciardi et al. (8)	Case report	1	Male	69	8	1	NR	Right	Right PPN	Pisa angle decreased from 52 to 30° within half a year after operation, then worsened
Artusi et al. (9)	Retrospective cohort	5	NR	NR	NR	NR	NR	NR	BL STN	Better
Roediger et al. (10)	Retrospective cohort	2	NR	NR	NR	NR	NR	NR	BL STN	Pisa angle decreased from 16.9 ± 2.0 to 5.5 ± 4.7°
Umemura et al. (11)	Retrospective cohort	10	8 female/2 male	NR	11.5	NR	NR	NR	BL STN	5 improved after DBS, 2 improved in follow-up, 3 had no improvement in both short term and long term after DBS

*BL, bilateral; Gpi, globus pallidus internus; PPN, pedunculopontine nucleus; STN, subthalamic nucleus; NR, not reported*.

The pathophysiology underlying Pisa syndrome is complex and multifactorial. The various hypotheses can be broadly classified into “central” versus “peripheral.” Central hypotheses refer to basal ganglia dysfunction and abnormal integration of sensory information. Peripheral hypotheses advocate an alteration of the musculoskeletal system, such as myopathy of the paraspinal muscles. The key role of asymmetry of basal ganglia outflow of central hypotheses is supported by both animal studies and clinical data (5). Therefore, it is reasonable to speculate that deep brain stimulation is likely to achieve improvement in lateral trunk deviations by correcting imbalances in basal ganglia outflow.

It has become widely accepted that in PD, the extent of pre-operative levodopa responsiveness predicts the extent of responsiveness to STN-DBS. In other words, DBS ameliorates symptoms that respond to dopaminergic medications (12, 13). Schlenstedt (4) reported that responsiveness of UPDRS III and UPDRS Posture Item 28 to levodopa as independent variables in a linear regression model with the DBS effect on postural abnormalities. In contrast, some publications showed that the axial symptoms of PD unresponsive to levodopa were ameliorated by bilateral STN stimulation (14). In case 2 we reported, lateral trunk flexion was not improved with 1.5 times the regular levodopa dose before DBS. However, this patient obtained a reduced Pisa angle from 14 to 6° in the process of post-operative programming. A study from Umemura (11) obtained similar results as ours. Umemura reported the effect of STN-DBS on postural abnormality in 18 PD patients (8 cases of camptocormia, 10 cases of Pisa syndrome) and 8 of them had poor pre-operative levodopa responsiveness. Four of these eight patients who had poor pre-operative levodopa responsiveness got good outcome on postural abnormality over a long period after surgery. This suggests that the pre-operative levodopa responsiveness on PS was not always consistent with the effect of DBS.

In addition, both patients in this study were characterized by a young onset (39 and 46 years, respectively) and a short period of time from the onset of Pisa syndrome to DBS surgery (0.5 and 2 years, respectively). Previous studies have shown that the occurrence of PS was associated with older age, higher levodopa equivalents, and more severe dyskinesia (1, 3). Also, short time of trunk flexion, mild severity, and good response to dopaminergic drugs are predictors of the efficacy of DBS in the treatment of camptocormia (9, 11). Therefore, the better outcome observed in our study may be related to the aforementioned reasons. Also, conclusions cannot be drawn with so few patients. We will continue to focus on this issue with more patients in the future.

## Conclusion

In summary, the lack of unified diagnostic criteria, incomplete data, data mostly from retrospective studies and case reports, as well as the complexity of its own mechanism have greatly led to the lack of understanding of the pathophysiological mechanism of patients with Parkinson's disease with PS, and then affected the development of its treatment. The establishment of more prospective studies, the use of more scientific technical means to record the data of posture abnormalities before and after treatment, and the increase of imaging, EMG, and other auxiliary examination methods may be more important for us to better understand PS and take more appropriate treatment measures to alleviate the posture abnormalities of PD patients in the future.

## Data Availability Statement

The raw data supporting the conclusions of this article will be made available by the authors, without undue reservation.

## Ethics Statement

The studies involving human participants were reviewed and approved by Ethics Committee of Beijing Hospital. The patients/participants provided their written informed consent to participate in this study.

## Author Contributions

JH: data curation, investigation, and writing—original draft preparation. ZC: surgical intervention. SL: methodology and investigation. HC: writing—reviewing and editing. WS: conceptualization and funding acquisition. All authors contributed to the article and approved the submitted version.

## Funding

The study was supported by Beijing Hospital Clinical Research 121 Project (121-2016009).

## Conflict of Interest

The authors declare that the research was conducted in the absence of any commercial or financial relationships that could be construed as a potential conflict of interest.

## Publisher's Note

All claims expressed in this article are solely those of the authors and do not necessarily represent those of their affiliated organizations, or those of the publisher, the editors and the reviewers. Any product that may be evaluated in this article, or claim that may be made by its manufacturer, is not guaranteed or endorsed by the publisher.

## Supplementary Material

Supplementary Video 1 Anatomical electrode location of case 1, electrodes were localized using lead-DBS software (http://www.lead-dbs.org).
